# Control of the interaction strength of photonic molecules by nanometer precise 3D fabrication

**DOI:** 10.1038/s41598-017-16496-x

**Published:** 2017-11-28

**Authors:** Colin D. Rawlings, Michal Zientek, Martin Spieser, Darius Urbonas, Thilo Stöferle, Rainer F. Mahrt, Yuliya Lisunova, Juergen Brugger, Urs Duerig, Armin W. Knoll

**Affiliations:** 1 IBM Research-Zurich, Rüschlikon, Switzerland; 2 SwissLitho AG, Technoparkstrasse 1, Zurich, Switzerland; 3 Microsystems Laboratory, Swiss Federal Institute of Technology Lausanne (EPFL), Lausanne, Switzerland

## Abstract

Applications for high resolution 3D profiles, so-called grayscale lithography, exist in diverse fields such as optics, nanofluidics and tribology. All of them require the fabrication of patterns with reliable absolute patterning depth independent of the substrate location and target materials. Here we present a complete patterning and pattern-transfer solution based on thermal scanning probe lithography (t-SPL) and dry etching. We demonstrate the fabrication of 3D profiles in silicon and silicon oxide with nanometer scale accuracy of absolute depth levels. An accuracy of less than 1nm standard deviation in t-SPL is achieved by providing an accurate physical model of the writing process to a model-based implementation of a closed-loop lithography process. For transfering the pattern to a target substrate we optimized the etch process and demonstrate linear amplification of grayscale patterns into silicon and silicon oxide with amplification ratios of ∼6 and ∼1, respectively. The performance of the entire process is demonstrated by manufacturing photonic molecules of desired interaction strength. Excellent agreement of fabricated and simulated structures has been achieved.

## Introduction

Device performance can frequently be significantly improved by moving from the typically 2D fabricated structure to a 3D geometry, for example in the case of plasmonic nanostructures^1^ or multi-gate (gate-all-around) FET transistors^2^. In the case of multigate FET transistors, the 3D profiles are achieved by transforming the 2D lithography masks into the desired profiles using pattern-transfer technologies such as dry etching. However, the use of a 2D lithography imposes restrictions on the achievable device geometries and requires careful development of the transfer processes.

**Figure 1:**
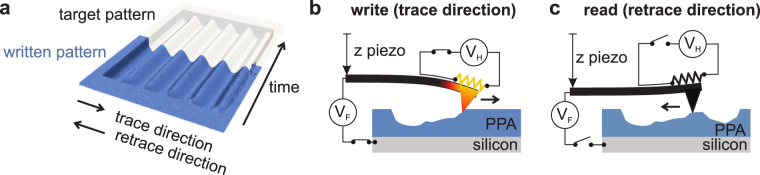
Fabrication of a 3D surface profile using a raster-scanned thermal probe to locally remove the resist material. (**a**) Target (gray) and actual experimental (blue) 3D surface profile formed by raster scanning the tip. (**b**) Write mode in trace direction: a voltage *V*_*H*_ is applied to the resistor located at the base of the cantilever’s tip to heat it using Joule heating. At each write pixel location, a voltage pulse *V*_*F*_ is applied to the substrate. This voltage generates an electrostatic force which pulls the tip from a distance of ≈100 nm into contact with the resist. The resist is evaporated as the tip moves in the trace direction. (**c**) Read mode (retrace direction): the heater and substrate voltages are switched off and the *z*-piezo is extended to bring the tip into contact with the surface. The patterning result is measured as the tip moves in the retrace direction. A feedback loop compares the target and the measured topography for the last read-back line and determines an appropriate update for the patterning parameters.

3D lithography removes the need for such time-consuming process development by directly defining the desired 3D structure. Grayscale lithography, i.e., the formation of a 3D surface profile (see Fig. 1a) is less flexible than fully 3D techniques, such as 3D direct laser writing^3^. However, grayscale lithography is directly compatible with the standard deposition and etching processes which are applied to the top of a sample following lithography, and thus has a wide range of applications. In optics, for example, it can be used to fabricate curved, multi-mode waveguides^4^ and optical cavities with high ratios of the Q-factor to the mode volume^5^ for quantum photonics^6–8^. In the field of electron optics, a helical structure could be used to impart orbital angular momentum to free electrons^9^. In contrast to the curved, multimode waveguide of ref.^4^, the latter examples require lateral resolutions on the order of a few 10 s of nanometers and nanometer precision in the *z*-direction^10^.

Electron beam lithography (EBL), the current state-of-the-art direct-write technique, is capable of such grayscale lithography. However, for feature sizes below 1 *μ*m, 3D proximity correction is required^11^, which demands careful calibration and can be computationally intensive even for line features^12^. Focussed ion beams (FIB) can also be used for grayscale lithography. However, the surface modification caused by the incident ions extends over several^13^ 100’s of nm and requires complex calculation of the “proximity-corrected” dose^14^. Moreover, FIB-milled surfaces are sensitive to further processing because of the damage caused by the incident ions^15^.

Thermal Scanning Probe Lithography (t-SPL) has demonstrated the capability to create grayscale patterns with high lateral and vertical resolution^16–19^. In t-SPL, a heated scanning probe is used to locally remove the resist polyphthalaldehyde, which decomposes endothermically when it comes into contact with a hot tip. As the resist exposure is localised to the dimensions of the tip^20^, long range proximity effects are absent. The write depth is controlled with high resolution by modulating the applied force on the cantilever. As a result, 3D grayscale patterns are fabricated in a single patterning run with high resolution^17^ rather than requiring one patterning run per depth level^16^. These patterns can be transferred into a target substrate by dry etching^16,21^.

For practical applications, however, high resolution is not sufficient, because it only provides high differentiation of depth levels locally. Both the total depth and the uniformity of nominally the same depth across the field size are typically inaccurate owing to imperfect calibration, minute tip changes and drift effects. These effects need to be mitigated for most real-world applications, which require absolute precision, termed accuracy, in the reproduction of depth values. In our previous work^17^, where these effects were not compensated, the vertical accuracy was limited to ±20 nm. Moreover, a stable and linear pattern transfer is required to faithfully achieve a similar depth control in a target substrate. Here we report on a solutions to both of these challenges which enable nanometer vertical accuracy in the transferred pattern. On the lithography side, we implement and expand the previously sketched concept^22^ of a feedback loop that is used to establish and maintain correct patterning parameters. We first outline and validate the model we use to describe the open-loop behavior. The model is then used in the framework of the Kalman algorithm to achieve a high-performance closed-loop lithography process. Next, we describe and characterize an enhanced transfer process, and demonstrate linear amplification and nanometer-scale control of the patterning depths in a silicon or silicon-oxide substrate. Finally, we fabricate wavelength-scale photonic molecules^23^. The fidelity of the entire fabrication process is demonstrated through a comparison of the cavities’ optical response with the response predicted by *ab initio* simulations performed for the target cavity dimensions.

## Results and Discussion

The concept of the closed-loop system relies on a combined write and read process as sketched in Fig. 1. The written lines, see Fig. 1b, are inspected during the patterning process by the tip as it returns to the start of the next write line, see Fig. 1c. The combined process assures that thermal drift between write and read pixels is negligible due to the short time difference of several milli-seconds. Thus, a fixed assignment between read and write pixels, and *in-situ* monitoring of the lithography process on a pixel-by-pixel basis become possible. The pattern depth information obtained is fed into a Kalman control algorithm to provide optimized patterning parameters for the next patterning line. The Kalman algorithm requires a model that relates the control or “state” parameters (cf. the patterning parameters) to the system’s observables (the patterning depth). This model must simultaneously be precise, to ensure fast convergence of the control loop, and computationally inexpensive so that it can be used during patterning. This paper begins by developing the model we use to relate the voltages applied to our cantilever (see Fig. 1b) to the written depth.

**Figure 2:**
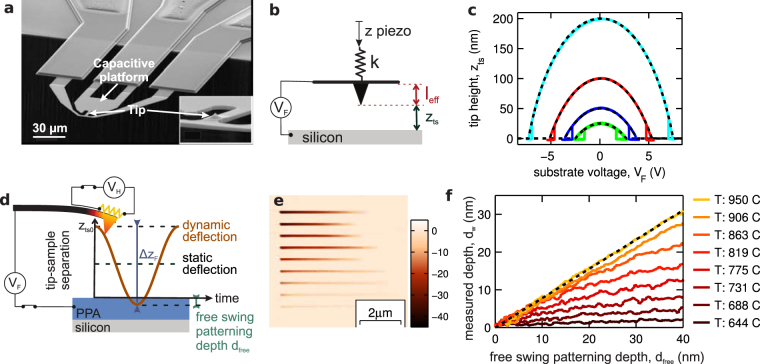
Experimental measurement of patterning dynamics. (**a**) SEM image of the t-SPL cantilever (**b**) Effective capacitor-spring system. (**c**) The cantilever’s tip height above the surface as a function of substrate voltage *V*_*F*_ for *z*_*ts*0_ = 25 nm, 50 nm, 100 nm and 200 nm. The experimental measurement for each height is shown by the solid line; the black dashed line is the result of solving eq. (1) for *l*_eff_ = 1.04 *μ*m, *εA*_eff_/2*k* = 3.9 × 10 ^−21^ Vm. (**d**) The static and dynamic deflections for the lightly damped cantilever system (Q ≃ 33). (**e**) t-SPL topography image after the patterning of a series of 114 nm wide lines, each written at a different heater temperature in a 120 nm thick PPA resist film. *V*_*F*_ has been steadily reduced from left to right. (**f**) The measured patterning depth for the center of each written line as a function of the free swing patterning depth. The curves have been shifted along the *x*-axis so that they pass through the origin (the unshifted curves are shown in Figure S3). The black dashed line has a slope of 0.8.

We first consider the capacitive actuation of our cantilever. The cantilever (see Fig. 2a) has in-plane dimensions of several 10 s of micrometers and a tip length of less than 1 *μ*m. Therefore the system can be modelled as a voltage supply *V*_*F*_ connected to a parallel-plate capacitor *C* whose upper electrode is constrained by a spring of stiffness *k* (see Fig. 2b). The tip-sample separation *z*_*ts*_ in the absence of a tip-sample force can be found by minimising the energy of such a system: $-\tfrac{1}{2}C({z}_{ts}){V}_{F}^{2}+\tfrac{1}{2}k{({z}_{ts}-{z}_{ts0})}^{2}$. Specifically, *z*_*ts*_ is obtained in terms of the system’s effective parameters from the real root $x=({z}_{ts0}-{z}_{ts})/({l}_{{\rm{eff}}}+{z}_{ts0})$ of the equation

1 $$x:{x}^{3}-2{x}^{2}+x={V}_{F}^{2}\frac{\varepsilon {A}_{{\rm{eff}}}}{2k{({z}_{ts0}+{l}_{{\rm{eff}}})}^{3}}$$

where A_eff_ is the effective area of the parallel plate capacitor, *ε* is the dielectric permittivity and *l*_eff_ is the effective length of the cantilever tip. *z*_*ts*0_ is the tip-sample separation in the absence of an applied voltage.

Figure 2c shows the measured tip height as a function of the substrate voltage *V*_*F*_ for a silicon substrate. This measurement is possible because the cantilevers have integrated thermoresistive distance sensors^24^, which, in contrast to a conventional optical detection setup, can measure the absolute tip-sample separation. Results for four different z-piezo positions *z*_*ts*0_ are shown by the solid lines. The black dashed lines show the results of a simultaneous least-squares fit of equation (1) to the four experimental curves. The agreement of global model fit and data is excellent and also the measured values of *l*_eff_ = 1.04 *μ*m and $\sqrt{{A}_{{\rm{eff}}}}\mathrm{=16.3}\,\mu$m agree reasonably with the dimensions of the system. This suggests that equation (1) can be used to describe the cantilever’s static deflection accurately.

During patterning, the substrate voltage is switched by means of a solid-state relay on a timescale that is short in comparison with the mechanical time constants for the cantilever. Therefore dynamic effects cannot be neglected. Representative values for the static and dynamic behaviour of our optimised, stiffer (cf. *k* = 0.065 Nm ^−1^ in ref.^17^) cantilevers are *k* = 0.3 Nm^−1^, *f*_*R*,1_ = 135 kHz and Q = 33. Ignoring the non linear nature of the electrostatic force, the peak dynamic deflection for a given *V*_*F*_ will be a factor of $R=\mathrm{2(1}-\pi \mathrm{/4}{\rm{Q}})=1.95$ times greater than the static deflection (see Fig. 2d). Thus the peak cantilever deflection Δ*z*_*F*_ due to the substrate voltage *V*_*F*_ will be

2 $${\rm{\Delta }}{z}_{F}({V}_{F})=R({z}_{ts0}-{z}_{ts}({V}_{F}\mathrm{)).}$$

This peak deflection is obtained roughly 4 *μ*s after *V*_*F*_ is switched on. In our system, the voltage pulse is therefore applied for 5 *μ*s so that the cantilever executes a single swing for each write pixel.

In write mode, the tip is heated via a resistive, low-doped region located at the base of the tip (see Fig. 1b). The relationship between the heater voltage *V*_*H*_ and the resistor temperature *T*(*V*_H_) can be calibrated by first measuring the current-voltage characteristic for the heater^24^ (see Figure S1). This heating of the cantilever results in a thermal deflection due to a material asymmetry introduced during the fabrication process. This deflection can only be measured with a thermally insensitive substrate and is typically 150 nm at 950 °C (see Figure S2). This out-of-plane deformation is relatively small compared with the dimensions of the cantilever and can therefore be well described simply as an offset to the piezo’s *z* position Δ*z*_*th*_(*V*_*H*_). At high temperatures the thermally sensitive resist will decompose resulting in small tip sample forces as the tip tries to penetrate into the resist. In the absence of tip-sample forces, the patterning depth *d*_*w*_ will be equal to the free swing depth *d*_free_ (see Fig. 2d). *d*_free_ can be calculated from equation (2) as

3 $${d}_{{\rm{free}}}({V}_{F},{V}_{H})={z}_{ts0}-{\rm{\Delta }}{z}_{th}({V}_{H})-{\rm{\Delta }}{z}_{F}({V}_{F}\mathrm{).}$$

Figure 2e shows a t-SPL topography image recorded while writing 114- nm-wide lines into a PPA film. Each line was written at a different temperature between 640 °C and 950 °C, and the substrate voltage *V*_*F*_ was varied along the line. For each combination of *V*_*F*_ and *V*_*H*_, the free swing depth below the surface, *d*_free_, was calculated from equation (3). The tip-sample separation *z*_*ts*0_ used for writing was 300 nm. To highlight the material’s response, we have shifted each curve in Fig. 2f along the *x*-axis so that each one passes through the origin (the unshifted curves are shown in Figure S3).

Figure 2f shows that at temperatures above 900 °C the measured patterning depth *d*_*w*_ depends linearly on the free swing depth *d*_free_. Such that:

4 $${d}_{w}=m{d}_{{\rm{free}}}+{z}_{{\rm{offs}}}$$

where *m* is the slope of linear relationship and *z*_offs_ is the shift we apply to our calibration data to ensure that the curves pass through the origin. This shift is typically ±20 nm (see Figure S3). For *T*  = 950 °C (black dashed line, Fig. 2f), the slope of this line is slightly below unity (0.8), likely because of non vanishing, repulsive tip-sample forces. However, the assumption of a unity slope provides a relatively accurate initial estimate of the patterning dynamics. The roughness of the pattern reduces with increasing temperature. At lower temperatures, the statistical nature of the polymer decomposition process can lead to incomplete decomposition of material, which in turn would lead to the observed higher pattern roughness. For *T* = 950 °C, the root mean square (RMS) deviation from a line of best fit (black dashed line, Fig. 2f) is *Rq*_,tool_ = 0.4 nm. This roughness represents the intrinsic variability of our patterning process.

During patterning the heater voltage is kept constant, and the substrate voltage for each write pixel ${V}_{F}^{(i)}$ is calculated by first obtaining the required free swing depth from equation (4) and then using equation (3) (see supplementary information for further details) to calculate the corresponding substrate voltage. To write a pattern with sub-nanometer accuracy, the value of the offset *z*_offs_ and the slope *m* must be further refined. By writing in the trace direction and reading in the retrace direction (see Fig. 1) the system continuously performs a measurement of the written pattern depth ${d}_{w}^{(i)}$ as a function of target depth ${d}_{t}^{(i)}$ (see supplementary information for details). Cross-correlation is used to ensure that the read and write frames are correctly aligned. Once a fixed number (typically 100) of data points have been collected, the t-SPL tool performs a linear regression for this set of points $\{({d}_{w}^{(i)},{d}_{t}^{(i)})\}$. This calculation yields an estimate of the parameters ${z}_{{\rm{offs}}}^{\ast }$ and *m* ^*^ along with the measurement uncertainty for this estimate. Next the tool updates the values of *z*_offs_ and *m* it uses to calculate the substrate voltages *V*_*F*_. The size of this update depends on the measurement uncertainty and is determined using the Kalman algorithm^25^. This cycle of measurement and update is performed continuously during the write process.

**Figure 3:**
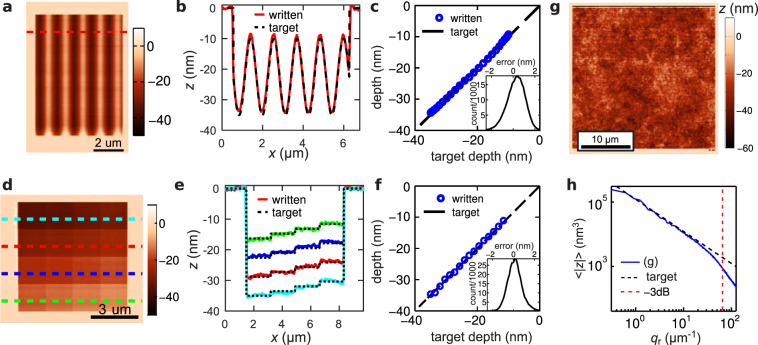
3D patterns written into a 110 nm thick PPA film to quantify the accuracy of the closed loop lithography process. Write pixel size was 11.4 nm, read pixel size was 5.7 nm. One write pixel was written every 15 *μ*s. (**a**) t-SPL topography image of a sine wave pattern written between depths of 10 nm and 35 nm. (**b**) Written (red line) and target depth (black dashed line) along the red cross section of (**a**). (**c**) Written vs target depth for each depth level in the upper 75% of the pattern in (**a**). The error distribution for all pixels is shown in the inset. (**d**) t-SPL topography image of a 4 × 4 chequerboard pattern with a level spacing of ≈1.7 nm. (**e**) The target (black dashed line) and written depths (colored solid lines) along the cross sections in (**d**). (**f**) Written vs target depth for each depth level in (**d**). The error distribution is shown in the inset. (**g**) Topography image of a self-affine surface roughness pattern. (**h**) Average spectral amplitudes $\langle |z|\rangle$ of (**g**) (blue line) compared to the target spectrum (red dashed line).

Figure 3 shows the result of using the closed-loop lithography process to write grayscale patterns. The result of writing a sine wave in a 110 nm thick film between depths of 10 nm and 35 nm is shown in Fig. 3a. The variation visible in the lower 10% of the pattern occurred as the feedback loop corrected for errors in the static calibration. Figure 3b shows the relationship between the written and target depths along the red cross section of Fig. 3a. The average written depth and the error distribution for the upper 75% of the pattern are shown in Fig. 3c. Figure 3d and e present the result of writing 16 tiles each at a constant target depth. The corresponding relationship between the written and the target depth is shown in Fig. 3f. The 1σ error was 0.85 nm for the sine wave pattern and 0.69 nm for the chequerboard pattern. These uncertainties compare favourably with the intrinsic roughness of a spin-cast polymer film of ≃0.5 nm^26^ and the feedback scheme introduces only a few angstroms of additional roughness to the intrinsic roughness of the patterning process (*R*_*q*,*tool*_ = 0.4 nm). Overall the patterning process achieves a fidelity that is compatible with the requirements of, for example, the spiral phase plate for imparting orbital angular momentum to free electrons^10^.

The power spectral density of the surface topography emerges as an important parameter^27,28^ in theories of contact mechanics and tribology. Using our closed-loop lithography process, it is possible to define an artificial surface “roughness” spanning a length scale from several 10 s of nanometers to several 10 s of micrometers. Figure 3g shows a self-affine artificial surface roughness with a $\mathrm{1/|}k|$ dependence of the amplitude on the wavenumber. This functional dependence is exhibited by spin-cast films^29^. Figure 3h show the analysis of the frequency content of the artificial roughness surface. For more than one order of magnitude in frequency space there is excellent agreement of the measured and the target amplitudes. The largest errors are present in the lowest frequency components. This is due to cross talk between the capacitive force used to actuate the lever and the written pattern. The polymer is a dielectric material, and for these larger patterns eventually sufficient material is removed so that the capacitance of the cantilever-sample system is affected. In Fig. 3h, it is also evident that errors emerge at higher frequencies due to the finite size of the probe used to fabricate the pattern. The written amplitude falls to half the target amplitude for a half wavelength of *λ*_*c*_ = 48 nm for Fig. 3g. This lateral resolution is consistent with the finite opening angle slope of the t-SPL tip and the patterning depth.

**Figure 4:**
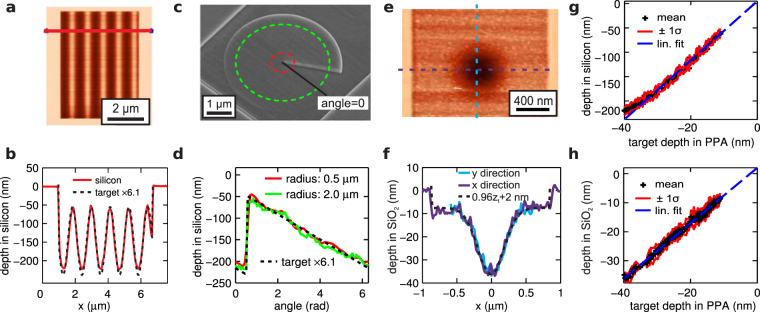
Result of transferring 3D patterns from the PPA resist layer into a substrate using reactive ion etching. (**a**) AFM topography image of a silicon surface after the transfer of a sine wave pattern. The pixel size was 39 nm. (**b**) Cross section along the read line in (**a**) compared to the target profile (black dashed line) scaled by a factor of 6.1. (**c**) SEM image of a spiral phase plate geometry. (**d**) The topography measured by the AFM and target profile along the circular paths shown in (**d**). The pixel size was 31 nm. (**e**–**f**) Results for the transfer of a 30 nm deep and 450 nm wide (full width half maximum) Gaussian cavity transferred into a sputtered silicon oxide layer. The pixel size for the AFM image was 12 nm. (**g**–**h**) The Distribution of measured depths in silicon (**g**) and silicon oxide (**h**) as a function of the target depths in PPA for the examples shown.

For many applications it is required to transfer the profile from the PPA resist into a silicon or silicon oxide layer, either to realise the pattern in a more robust material or to amplify the topography^16,21,30^. We optimized the transfer stack geometry by introducing a 3 nm thin PMMA layer between the substrate and the PPA for thermal isolation. The additional layer resulted in a reduction of the residual polymer layer when patterning to the maximium possible depth (writing with the largest possible heating voltage *V*_*H*_ and writing force *V*_*F*_) on silicon from ≈25 nm^31^ to ≈10 nm. This reduction in the residual polymer layer is important for high fidelity transfer. The improvement likely arose because the additional silicon-PMMA and PMMA-resist interfaces improve the isolation of the thermal resist from the silicon which acts as a heat sink. This heat sink counteracts the heating effect of the tip eventually preventing decomposition of the resist. Furthermore, we found appropriate etching parameters of our silicon dry etch^16^ to obtain a linear transfer of the grayscale patterns into the substrate (see methods section for details). In Fig. 4, we present the result of transferring several patterns relevant for applications from the PPA layer into a silicon or silicon oxide substrate. Figure 4a and b show the result of transferring a sine wave patterned important for distributed feedback devices into silicon using this method. Figure 4c and d show a fabricated spiral phase plate geometry^9,10^ in silicon. Figure 4e and f show equivalent results for the transfer of a Gaussian deformation with a width of 450 nm and a depth of 30 nm into a sputtered silicon oxide layer using the same etch process as for Fig. 4a–4d. All cross sections in Fig. 4 are compared to the absolute target depth programmed during patterning and amplified with the calibrated amplification factor. Only in the case of the silicon oxide transfer the pattern was slightly under-etched and an offset of 2 nm was added.

The distribution of depths in the etched sample has been calculated for each depth level appearing in the target pattern and is shown in Fig. 4g and h for silicon and silicon oxide, respectively. The transfer into silicon oxide yields a highly linear relationship (Fig. 4g) between the depth in the final silicon oxide structure and the target patterning depth in the PPA. In the case of the silicon transfer (Fig. 4b), the relationship is also highly linear for the target depth range from 35 nm to 10 nm. However, a deviation of 7% is visible for the deepest target depth. We believe that this is due to a non-linearity in the early phase of the polymer etch process due to the chemical modification of the etched polymer surface. For future work, a slightly thicker residual PPA layer could be used to ensure a linear transfer of the full range of written depths. The slope of the blue dashed line in Fig. 4b and g gives the amplification ratio for the pattern-transfer process. For this choice of etch chemistry we observe an amplification ratio of 6.1 for silicon and 0.96 for SiO_2_.

For the silicon pattern of Fig. 4a the average 1σ width of the distributions for each depth level is 8.4 nm. The written pattern used for the etching had a 1σ absolute error of 1.3 nm. The amplified roughness (6.1 × 1.3 nm) agrees to within 6% with the observed silicon roughness. This shows that the transfer does not introduce additional roughness into our patterns. The final RMS error in the silicon oxide structure is 1.8 nm. Comparison with an etch test on an unpatterned layer yielded a surface roughness of 1.3 nm. For transfer into silicon oxide the quality of our final structure is limited by the quality of the sputtered silicon oxide rather than the accuracy of the t-SPL patterning. This is in contrast to the situation when transferring into silicon.

**Figure 5:**
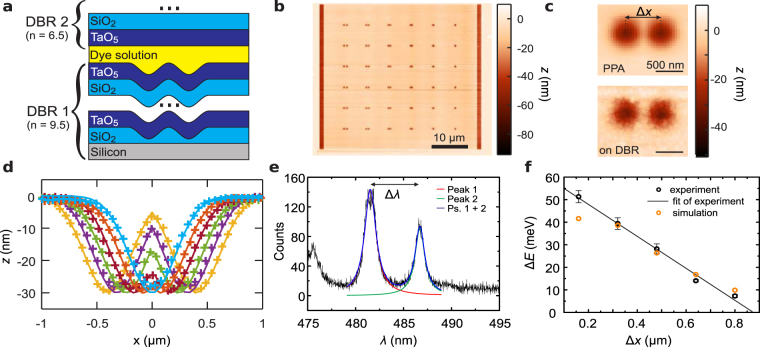
Fabrication and characterization of photonic molecules. (**a**) Schematics of the photonic stack. The patterns are fabricated in the bottom SiO_2_ layer before deposition of DBR 1. A dye solution (0.1% Coumarine in toluene) acts as optically active medium. (**b**) Topography of the t-SPL written pattern consisting of 6 × 6 pairs of 3D Gaussian patterns (**c**) Close-up of a twin Gaussian structure after t-SPL (top) and after transfer into SiO_2_ and DBR deposition. (**d**) Average Cross-sections (symbols) along the first lines of twin Gaussian structures in PPA for Δ*x* = 800, 640, 480, 320, 160, and 0 nm. Lines indicate the target pattern. (**e**) Fluorescence spectrum (black line) of a photonic molecule for Δ*x* = 480 nm separation of the Gaussians. Colored lines indicate Lorentzian fits. The spectral splitting Δλ is a measure for the coupling strength Δ*E* of the two microcavities. The peak at 476 nm corresponds to the unstructured part of the sample which acts as a planar Fabry-Perot cavity. (**f**) Δ*E* as a function of the distance between the Gaussian centers. The error bars are given by the standard deviation of the measurements of the 6 nominally identical devices. The solid line is a linear fit to the data to guide the eye. The orange symbols are the results from ab-initio numerical simulations.

A key area of application for high resolution grayscale patterning is nanophotonics, where structures of several hundred nanometers up to a few micrometers require fabrication with nanometer precision. Here we realize vertical microcavities, see Fig. 5a, with high quality factor and wavelength-scale mode volume by creating smooth Gaussian deformations (depth 30 nm, full-width at half-maximum 450 nm)^32^ (see Methods for details) that provide strong lateral confinement of the optical mode in addition to the longitudinal confinement between two distributed bragg reflectors (DBRs). Placing two identical Gaussians within a small distance on the order of the wavelength, a so-called photonic molecule can be formed, where the two zero-dimensional cavities are coupled through the evanescent fields. This coupling leads to a splitting of the mode into a “bonding” and an “anti-bonding” mode, which energy separation is set by the distance between the Gaussian deformations and crucially depends on the fabrication accuracy of the structures.

For statistics, we prepared for the six spacings ranging linearly from Δ*x* = 0 to Δ*x* = 800 nm six nominally identical cavities. Figure 5b shows the topography of the patterning field in PPA containing all 36 Gaussian pairs. After pattern transfer to silicon oxide the roughness increases, see Fig. 5c for a closeup of a Gaussian pair with Δ*x* = 640 nm before and after transfer. Figure 5d compares cross-sections through the center of the lowest row of structures (symbols) with the target patterns (lines).

Figure 5e shows the emission of a Gaussian pair with Δ*x* = 640 nm after addition of the two DBRs, filling with Coumarine solution, and optical excitation. A clear separation of two distinct Lorentzian shaped peaks is observed, attributed to the mode splitting Δ*E* of the system. Figure 5f shows the mode splitting, i.e. the coupling strength between the microcavities, as a function of the distance between the Gaussians’ centers. Comparing the measurements to *ab-initio* three-dimensional finite-difference time-domain (3D FDTD) simulations we find excellent agreement without any fit parameter (except for the smallest distance where the simulation becomes slightly inaccurate owing to memory limitations on the computational cell size). A similar accuracy could not be achieved using focused ion beam (FIB) milling for fabrication^8^. It is an important milestone for extending the basic building block of the photonic molecule towards larger arrays of microcavities which could be used for polariton quantum simulations of crystal lattices^33^ or quantum computing with trapped ions^34^.

In conclusion, we have developed a complete resist-patterning and pattern-transfer solution that provides high resolution lateral and nanometer-accurate vertical fabrication of grayscale patterns in a substrate. To achieve this goal, we have implemented a closed-loop lithography process that is capable of fabricating surface profiles in resist with an accuracy of less than 1 nm in a single pass. This required a thorough analysis of our open-loop system. First, we determined that our cantilever–substrate system could be approximated as a parallel-plate capacitor attached to a spring. Second, we found that material removal was governed by the dynamics of this mechanical cantilever system if sufficiently high temperatures were used. In this operating regime, our model provides an accurate description of the system and allows the implementation of a model-based control scheme capable of nanometer control over the absolute patterning depth. Moreover, we have shown that the transfer and amplification into silicon and silicon oxide proceed linearly such that the fidelity and accuracy of our written resist patterns are not compromised during the etch transfer.

We demonstrate the potential of our approach by fabricating wavelength-scale photonic molecules^23^ with a target geometry and, correspondingly, a target energy-mode splitting. We found excellent agreement of the measured optical response with simulations based on the target geometry. This capability is a key step to extend the concept of photonic molecules towards the fabrication of coupled microcavities of designed coupling strength, needed for quantum simulations of 2D lattices. Moreover, the result is further evidence of the accuracy of our fabrication approach and the precision to target the desired function in a nanoscale device. In general, we anticipate that the ability to fabricate 3D topographies with nanometer accuracy will open up new routes for the experimental realization of nanoscale devices as well as for exciting fundamental studies in fields ranging from tribology to electron optics.

## Methods

### Film preparation

The resist films for the patterns shown in Fig. 3 were prepared by spin coating a solution of polyphthalaldehyde dissolved in anisole. The 120- nm-thick films were spun at 4000 rpm from a 5% weight solution.

The films used for the silicon etching were prepared in three steps. First the wafer was cleaned by dipping it into buffered HF solution, followed by exposure to an oxygen plasma in a barrel asher. Then a 3-nm-thick PMMA layer was spin-coated from a 0.2% solution of PMMA in anisole. The PMMA solution was obtained by dilution of a 2% 950 PMMA resist solution supplied by Micro Chem. Finally, a 65-nm-thick PPA layer was spin-coated on top of the PMMA layer from a 3% weight solution at 2700 rpm. The film was baked at 90 °C for 3 minutes to remove the residual solvent.

### t-SPL patterning

The thermal scanning probe lithography (t-SPL) was performed on our home-made system, which has been described in detail elsewhere^16,17^. Details on the feedback scheme are provided in the supplementary information.

### Reactive ion etching

The reactive ion etching was performed using a deep reactive ion etching tool from Alcatel. SF_6_ was used in conjunction with C_4_F_8_ at flow rates of 40 sccm and 60 sccm, respectively. The source power was 1500 W and the acceleration power was 15 W.

### Fabrication and characterization of photonic molecules

The microcavity consists of two mirrors with distributed Bragg reflectors (DBR) with a quarter-wavelength stack of 6.5 and 9.5 layer pairs of Ta_2_O_5_/SiO_2_ from magnetron sputtering. For one mirror, we pattern the Si substrate (PPA transfer) with the Gaussian-shaped domes prior to the mirror deposition. For the other mirror, we define a ~200 *μ*m diameter mesa structure with 30 *μ*m back-etch on a borosilicate substrate to obtain a small mirror area with reduced sensitivity to surface defects or particles. Both mirrors are mounted within a micro-photoluminescence setup on different XYZ nanopositioning stages where the distance is controlled to change the resonance wavelength. In order to measure the optical modes in fluorescence, we drop cast 0.1% Coumarine in Toluene solution between the mirrors and use a 405 nm fiber coupled continuous wave laser focused to 2 *μ*m spot size for excitation. The light is collected by a microscope objective and analyzed in a fiber-coupled spectrograph. For the photonic simulations we employed 3D FDTD calculations^35^ with a spatial grid resolution of 4 nm, using the same targeted geometry of the two coupled Gaussians that served as input to the fabrication. The refractive indices for the DBR materials were obtained from ellipsometry (nTa_2_O_5_ = 2.115 and nSiO_2_ = 1.476).

## Electronic supplementary material

Supplementary information
