# Supplementary material for: Control of the interaction strength of photonic molecules by nanometer precise 3D fabrication
Source: Sci Rep. 2017 Nov 28;7:16502. doi: 10.1038/s41598-017-16496-x (PMC5705769; doi:10.1038/s41598-017-16496-x)
Supplement: Supplementary file 1 — Supplementary information [file 41598_2017_16496_MOESM1_ESM.pdf]

Supplemental Information: Control of the interaction strength of photonic molecules by nanometer precise 3D fabrication

Colin Rawlings[1], Michal Zientek[1], Martin Spieser[1,2], Darius Urbonas[1], Thilo Stöferle[1], Rainer F. Mahrt[1], Yuliya Lisunova[3], Juergen Brugger[3], Urs Duerig[1], and Armin W. Knoll[1,*]

[1]IBM Research - Zurich, Rüschlikon, Switzerland
[2]SwissLitho AG, Technoparkstrasse 1, Zurich, Switzerland
[3]Microsystems Laboratory, Swiss Federal Institute of Technology Lausanne (EPFL), Lausanne, Switzerland
[*]ark@zurich.ibm.com

Contents

1 Calibration of cantilever **2**
1.1 Writer temperature 2
1.2 Thermal deflection of Cantilever 2
1.3 Open loop patterning performance 4

2 Closed Loop Lithography Scheme **5**
2.1 Kalman Filter 5
The Model
2.2 Calibration Parameters 5
2.3 Patterning 5
2.4 Observables 6
2.5 Application of the Kalman Algorithm 6
2.6 Additional considerations 7

References **7**

A Quadratic approximation to the parallel plate model **8**

1 Calibration of cantilever

1.1 Writer temperature

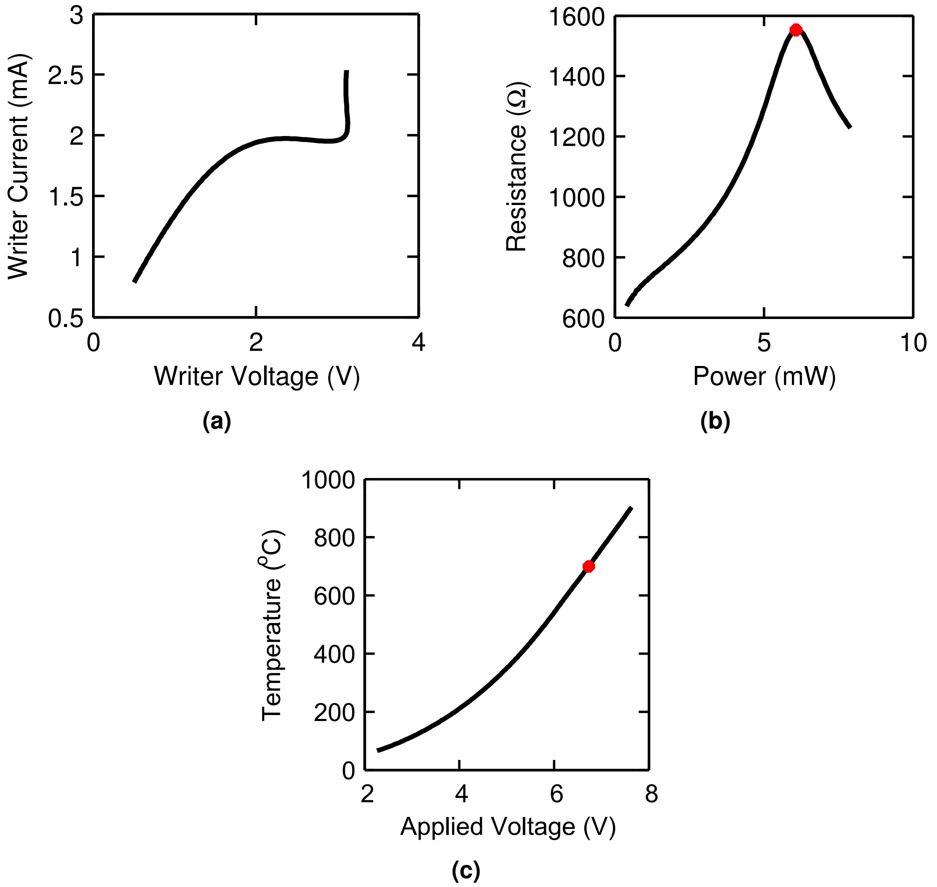

Figure S1. Measured electrical characteristics of the cantilever's write resistor. The applied voltage is connected to the write leg of the cantilever via a 1.5kΩ series resistance. The measurement was performed with the cantilever positioned 400 nm from the surface. (a) Current-voltage characteristic for write resistor (b) Resistance-power characteristic, the maximium resistance is marked by the red dot. For the writer's doping density of 1.5×10^{18} this corresponds to a temperature of $\simeq$700°C. (c) Calculated dependence of writer temperature on applied voltage for the cantilever.

The cantilever's tip is heated by means of a low doped region located at its base. The relationship between writer temperature and applied voltage is determined from a measurement of its current-voltage characteristic (see figure S1a). The resistivity exhibits a maximum which depends on the dopant density of the resistive region[1]. The power dissipated at this point of maximium resistance can be found by calculating the resistance-power characteristic (see figure S1b). Based on the assumption that the temperature varies linearly with power the relationship between applied voltage and temperature can finally be calculated (see figure S1c).

1.2 Thermal deflection of Cantilever

It has been observed experimentally that the cantilevers deform when heated by the writer. This deformation is relatively consistent between cantilevers from a given wafer and cantilevers always deform in the direction pointed by the tip. The effect likely arises from a material asymmetry introduced during fabrication. It is difficult to measure the effect at high temperatures when operating on a PPA resist as the resist decomposes when the tip contacts the surface. However, it may be measured on a thermally insensitive substrate such as silicon before commencing patterning.

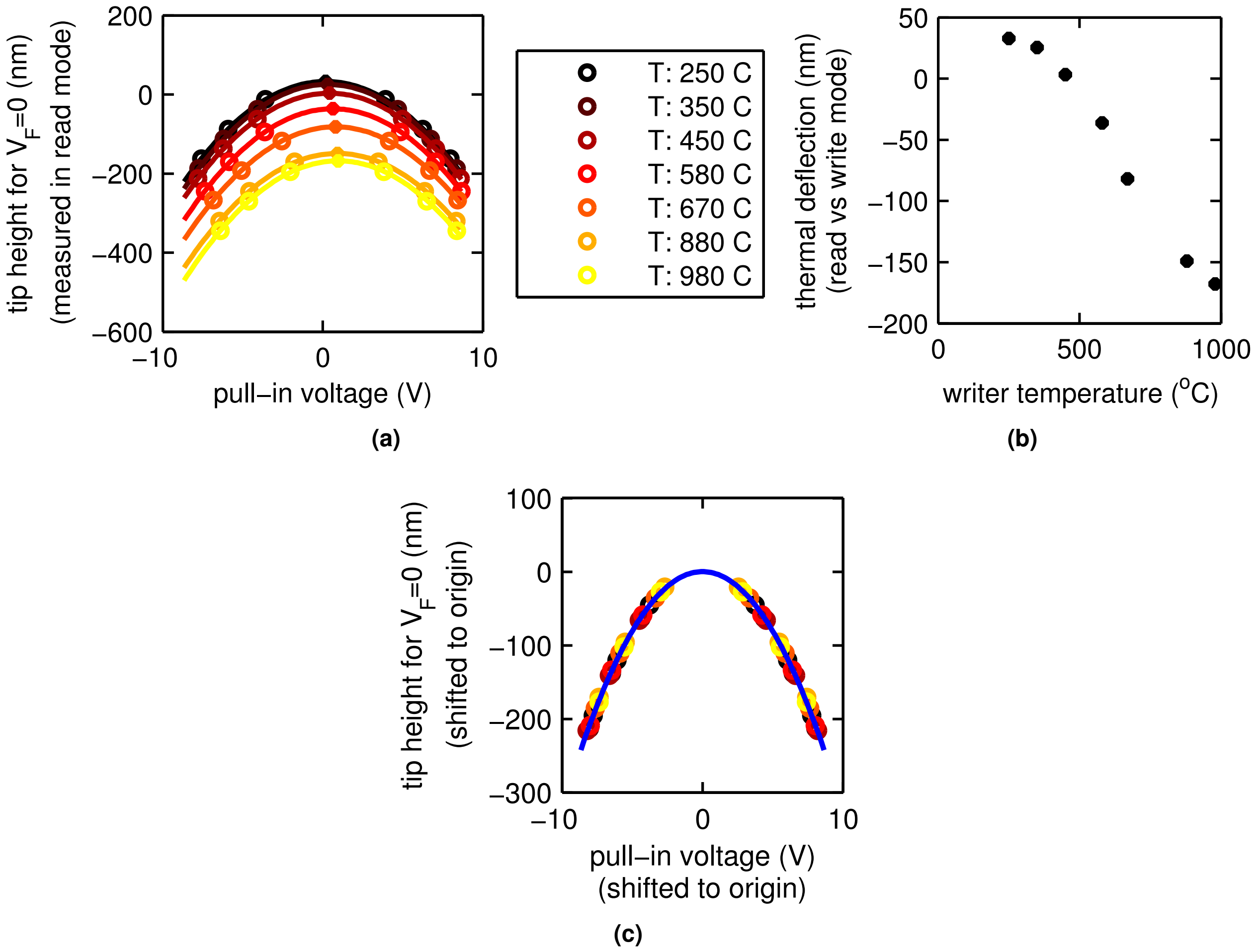

Figure S2. Measurement of the cantilever's thermal deflection as a function of temperature. (a) The separation between tip and sample set using the piezo when the cantilever is in read mode and the substrate voltage (V_F) =0 is plotted on the y axis. The voltage required to pull the tip onto the surface after switching to write mode is displayed on the x axis. Measurements for a range of cantilever temperatures are shown by the circles. The solid line shows a least squares fit of a parabola to the data. (b) The thermal deflection of the cantilever inferred from the position of the maxima of the curve in (a) as a function of temperature. (c) The result of shifting the curves in (a) so that the maxima lies at the origin.

The measurement can be performed either by activating the writer and approaching the surface with the piezo or by fixing the piezo position close to the surface and using the substrate voltage to pull the tip into contact. Because the writer signal is of a lower quality particularly at temperatures close to the resistivity maximium we perform the latter measurement. This avoids that the tip-sample contact is missed by the control software such that the piezo drives the tip into the sample.

With the cantilever in read mode the piezo position corresponding to first tip-sample contact is measured and recorded. The piezo is then retracted by a fixed amount, the writer is activated and the substrate voltage required to pull the tip into contact with the surface is recorded. Contact is determined from the writer signal. Results for the cantilever are shown in figure S2a. From equation 1 it can be determined that the voltage $V_{F,contact}$ required to set $z_{ts} = 0$ is:

$$z_{ts0} = \alpha V_{F,contact}^2, \quad \alpha = \frac{\varepsilon A_{eff}}{2kl_{tip}^2} \tag{S1}$$

It can be seen that the data for each temperature is well fitted by a parabola.

The maxima for each temperature curve gives the thermal deformation of the cantilever. These values are plotted in figure S2b. At low writer temperatures ($T < 350°C$) the cantilever distortion in read mode exceeds that occuring in write mode leading to a positive value for the thermal deflection. The curves for each temperature shown in figure S2a may be replotted with their maxima shifted to the origin (see figure S2c). From this figure it can be seen that the shape of the curve does not change. This supports the assertion that the thermal deflection may be treated purely as an offset to the cantilever's height.

1.3 Open loop patterning performance

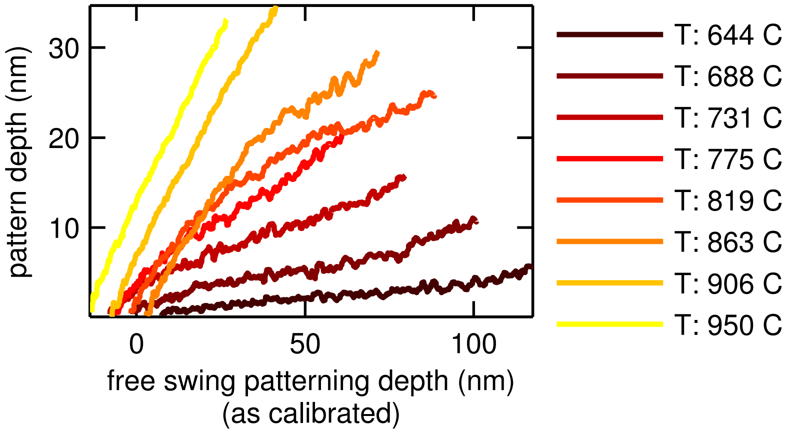

Figure S3. Unshifted curves corresponding to figure 2e and 2f

In figure 2f the cross sections for figure 2e were shifted so that they passed through the origin. Figure S3 shows the result of applying the calibration data of figures 2d and S2b to the free swing or nominal patterning depth as a function of measured patterning depth. At low temperatures the slope of the curves is well below 1. The offset of the curves for zero patterning depth is predicted to roughly ±20 nm.

2 Closed Loop Lithography Scheme

2.1 Kalman Filter

2.1.1 The Model

In this section we employ a quadratic approximation to the roots of equation 1. As shown in appendix A this approximation is quite reasonable over typical patterning depths. It allows for a presentation of results in closed form. Furthermore its simplicity is an asset when implementing the control scheme in real time in the face of limited computational resources.

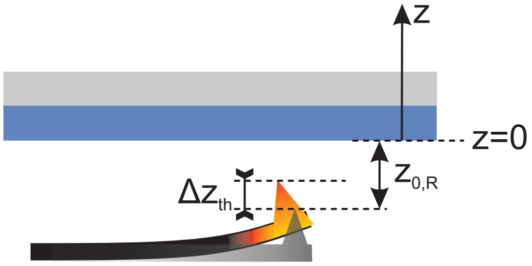

Figure S4. Sign convention used in the explanation of the Kalman filter implementation

We define written depths as positive numbers. The separation between the tip and sample $z_{0,R}$ (typical $z_{0,R} = -300\,\text{nm}$) is set with the cantilever in read (see figure S3). Activating the writer results in a thermal deflection Δz_{th} (see figure S2b) towards the surface. In the quadratic approximation applying the substrate voltage V_F would result in a static deflection of αV_F^2 where α may be obtained from equation (S1). Because of the light damping of the cantilever it overshoots the static deflection by an amount R$\simeq$ 1.95 when the voltage is applied abruptly. Thus in the absence of tip sample forces the end of the tip reaches a z position:

$$z_{w,free} = z_{0,R} + \Delta z_{th} + R\alpha V_F^2 \tag{S2}$$

assuming some phenomological ratio m between the actual patterning depth z_w and $z_{w,free}$ yields finally:

$$z_w = m(z_{0,R} + \Delta z_{th} + R\alpha V_F^2) \tag{S3}$$

2.2 Calibration Parameters

Following the calibration outlined previously the scan computer has estimates of the parameters appearing in equation (S3) which we denote with a tilde. The exception to this is $\tilde{m}$ which is assumed to be 1. Hence:

$$z_{w,nom} = \tilde{z}_{0,R} + \tilde{\Delta} z_{th} + \tilde{R}\tilde{\alpha} V_F^2 \tag{S4}$$

These parameters are not changed during the write process (although the physical values may change). Instead a pair of additional parameters are incorporated into the calculation of the write depths. These are p_0 and p_1:

$$z_{w,nom} = p_1\left((z_w - \bar{z}_t) + p_0\right) \tag{S5}$$

These two parameters are used to correct the net effect of the difference between the true parameters and the parameters measured during the calibration. The offset $\bar{z}_t$ is the average target depth appearing in the written pattern. Shifting p_0 in this way helps to orthogonalise the system. For this choice of the p_i p_0 defines the average depth of the pattern while p_1 defines the spread of written depths.

2.3 Patterning

During patterning the write voltage for the ith pixel is calculated by substituting equation (S5) into (S4) to obtain:

$$V_F^{(i)2} = \frac{p_1(z_t^{(i)} - \bar{z}_t + p_0) - \tilde{z}_{0,R} - \Delta\tilde{z}_{th}}{\tilde{R}\tilde{\alpha}} \tag{S6}$$

This can be substituted into the (by assumption) correct patterning model (equation (S3)) to obtain the written depth for this pixel:

$$z_w^{(i)} = s_1 p_1 (z_t^{(i)} - \bar{z}_t) + s_0 + s_1 p_0 p_1 + n^{(i)} \tag{S7}$$

$$\text{where}: \quad s_1 = \frac{mR\alpha}{\tilde{R}\tilde{\alpha}} \tag{S8}$$

$$s_0 = (mz_{0,R} - s_1 \tilde{z}_{0,R}) + (m\Delta z_{th} - s_1 \Delta\tilde{z}_{th}) \tag{S9}$$

where $n^{(i)}$ is a random variable. This random component was measured experimentally and referred to as $R_{q,tool}$ in the text. From the point of view of the Kalman filter it is assumed to be drawn from a Gaussian distribution:

$$n^{(i)} \sim \mathcal{N}(0, R_{q,tool}) \tag{S10}$$

2.4 Observables

The set of written pixels $\{(z_t^{(i)} - \bar{z}_t, z_w^{(i)})\}$ are analysed using linear regression which yields a slope r_1 and an offset r_0. Again in the interests of orthogonality we define our observables as:

$$b_0 = r_0 + p_0 \tag{S11}$$

$$b_1 = r_1 \tag{S12}$$

Based on equation (S7) the expected value of r_0 is:

$$E[r_0] = s_0 + s_1 p_0 p_1 \tag{S13}$$

If the algorithm is close to estimating the state correctly $s_1 p_1 \simeq 1$. So that:

$$b_0 = s_0 + (s_1 p_1 - 1) p_0 \simeq s_0 \tag{S14}$$

or:

$$\begin{bmatrix} b_0 \\ b_1 \end{bmatrix} \simeq \begin{bmatrix} 1 & 0 \\ 0 & p_1 \end{bmatrix} \begin{bmatrix} s_0 \\ s_1 \end{bmatrix} \Rightarrow \mathbf{b} = \mathrm{H}\mathbf{s} \tag{S15}$$

The variance in the observables may be estimated from the variances estimated during the linear regression since:

$$\mathrm{Var}[b_0] = \mathrm{Var}[r_0], \quad \mathrm{Var}[b_1] = \mathrm{Var}[r_1] \tag{S16}$$

2.5 Application of the Kalman Algorithm

The initial or *a priori* estimate of the state vector is:

$$\mathbf{s} = \begin{bmatrix} 0 \\ 1 \end{bmatrix} \tag{S17}$$

Based on our experience we may select the initial values the terms in the diagonal matrix P describing the uncertainties in s_i as well as the process noise matrix S.

Our model does not assume any systematic variation of the state parameter so our state transition model A is simply the 2×2 identity matrix. We scale the measured uncertainties in our observables (b_0 and b_1) to reduce the size of the Kalman gain. During testing we found that this yielded more accurate written patterns albeit at the expense of slower convergence, i.e. greater errors in the first few written lines. The updated estimate of the state $\mathbf{s}_{k+1}$ is calculated from the well known Kalman equation[2] which seeks to minimise the expectation of the squared error in the elements of the state vector following a linear update step:

$$\mathbf{s}_{k+1} = \mathbf{s}_k + \mathrm{K}_k(\mathbf{b}_k - \mathrm{H}_k \mathbf{s}_k) \tag{S18}$$

$$\text{where}: \quad \mathrm{K}_k = \mathrm{P}_k \mathrm{H}_k^T (\mathrm{H}_k \mathrm{P}_k \mathrm{H}_k^T + \mathrm{R}_k)^{-1} \tag{S19}$$

likewise we update[2] P_k as:

$$\mathrm{P}_{k+1} = (1 - \mathrm{K}_k \mathrm{H}_k) \mathrm{P}_k \tag{S20}$$

2.6 Additional considerations

Our write pixel size is necessarily smaller than our tip size to ensure a smooth written pattern. Consequently write pixels on subsequent lines overlap with those on the line before. To ensure we correctly measure the final pattern we move backwards along the slow scan direction 100 nm before reading a line to ensure that the line being read is not subsequently modified. This means that we can only perform a Kalman update once 100 nm of write lines have been produced with the updated state vector and we finally start to observe write pixels with the new conditions. This reduces the speed at which our feedback algorithm can operate but does not otherwise compromise its stability or accuracy.

We supplement the Kalman filter with several "common sense" checks on the input data to the linear regression. For example points where the written depth is zero cannot be used and if all observed written depths are zero we simply increase p_0 by a pre-defined step. Likewise if all target depths for the given sample are the same then we only apply the Kalman filter to s_0 and remove s_1 from consideration until patterning data for distinct depth levels becomes available.

Stabilisation of the write height is an important additional consideration for achieving precise depth control. Any errors in the tip sample separation $z_{R,0}$ will appear directly in the pattern. We implement sub nanometer control of $z_{R,0}$ using the following procedures. Firstly after loading a sample we use the thermoresistive distance sensor to measure the sample tilt, which is typically on the order of 1 nm/μm. To measure the tilt the tip is retracted 100 nm from the sample and the sample is then scanned in first the x and then the y direction. The change in tip-sample separation during this motion is recorded and the tilt is calculated from this signal. This tilt is then used when calculating the scan trajectory. During scanning the extension of the z piezo changes as the tip moves between the read and write heights. To ensure this motion is accurate we employ a feed forward scheme[3]. We have observed that the z position of our tSPL system drifts by several nanometers during the patterning process, possibly because of heating of the piezos. To compensate for this drift we measure the change in reader signal during the retract and approach to the surface occuring when switching between the read and write heights. From these signals the absolute height of the system can be determined and the drift in $z_{R,0}$ accounted for.

References

1. U Duerig. Fundamentals of micromechanical thermoelectric sensors. *J. Appl. Phys.*, 98(4):044906–044906, 2005.
2. Robert Grover Brown. *Introduction to Random Signal Analysis and Kalman Filtering*. John Wiley and Sons, 1983.
3. Philip C Paul, Armin W Knoll, Felix Holzner, Michel Despont, and Urs Duerig. Rapid turnaround scanning probe nanolithography. *Nanotechnology*, 22:275306, 2011.

A Quadratic approximation to the parallel plate model

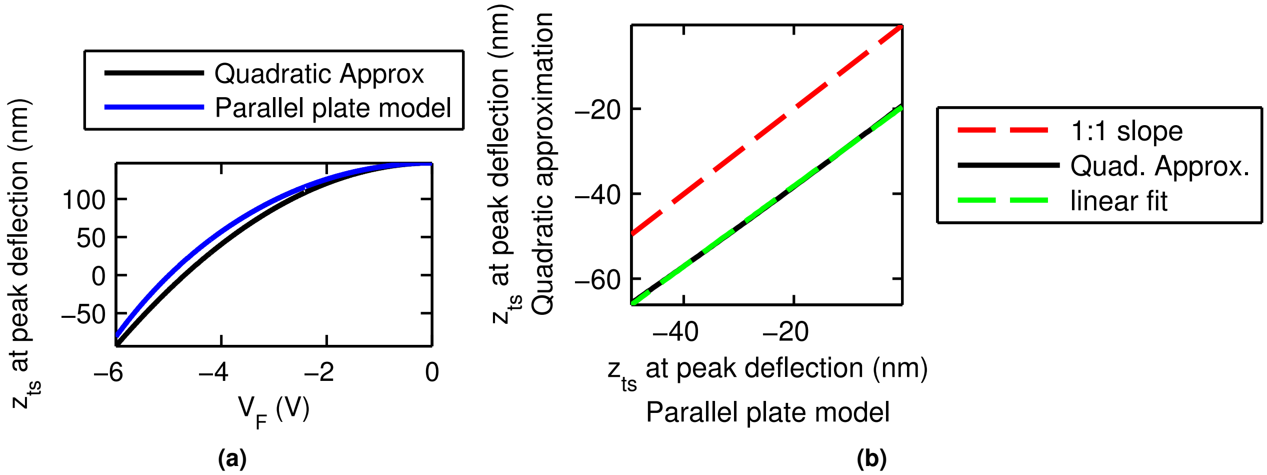

Figure S5. Comparison between maximium write depths calculated using the parallel plate model (eq 1) and the quadratic approximation of equation (S1). The cantilevers parameters were obtained from figure 2d. The write height was taken to be 300 nm. The thermal deflection was taken from figure S2b for a write temperature of 900°C. The dynamic overshoot was taken to be 1.95. (a) Calculation of maximium cantilever deflection using the parallel plate model and the quadratic approximation. (d) Comparison between calculated depth for the two methods for a write depth between 0 nm and 50 nm.

The accuracy of the quadratic approximation of equation (S1) may be assessed by calculating the deflections for the two methods for the same voltage and typical parameters. As can be seen in figure S5 the quadratic approximation introduces sub-nanometer errors due to non-linearity into the written depth. It introduces an offset in written depths of roughly 20 nm and a slope error of 5%.
